# Supplementary material for: Upregulation of Protein Synthesis and Proteasome Degradation Confers Sensitivity to Proteasome Inhibitor Bortezomib in Myc-Atypical Teratoid/Rhabdoid Tumors
Source: Cancers (Basel). 2020 Mar 22;12(3):752. doi: 10.3390/cancers12030752 (PMC7140067; doi:10.3390/cancers12030752)
Supplement: Supplementary file 1 [file cancers-12-00752-s001.pdf]

cancers

Article

Upregulation of Protein Synthesis and Proteasome Degradation Confers Sensitivity to Proteasome Inhibitor Bortezomib in Myc-Atypical Teratoid/ Rhabdoid Tumors

Huy Minh Tran, Kuo-Sheng Wu, Shian-Ying Sung, Chun Austin Changou, Tsung-Han Hsieh, Yun-Ru Liu, Yen-Lin Liu, Min-Lan Tsai, Hsin-Lun Lee, Kevin Li-Chun Hsieh, Wen-Chang Huang, Muh-Lii Liang, Hsin-Hung Chen, Yi-Yen Lee, Shih-Chieh Lin, Donald Ming-Tak Ho, Feng-Chi Chang, Meng-En Chao, Wan Chen, Shing-Shung Chu, Alice L. Yu, Yun Yen, Che-Chang Chang and Tai-Tong Wong

Supplementary Materials

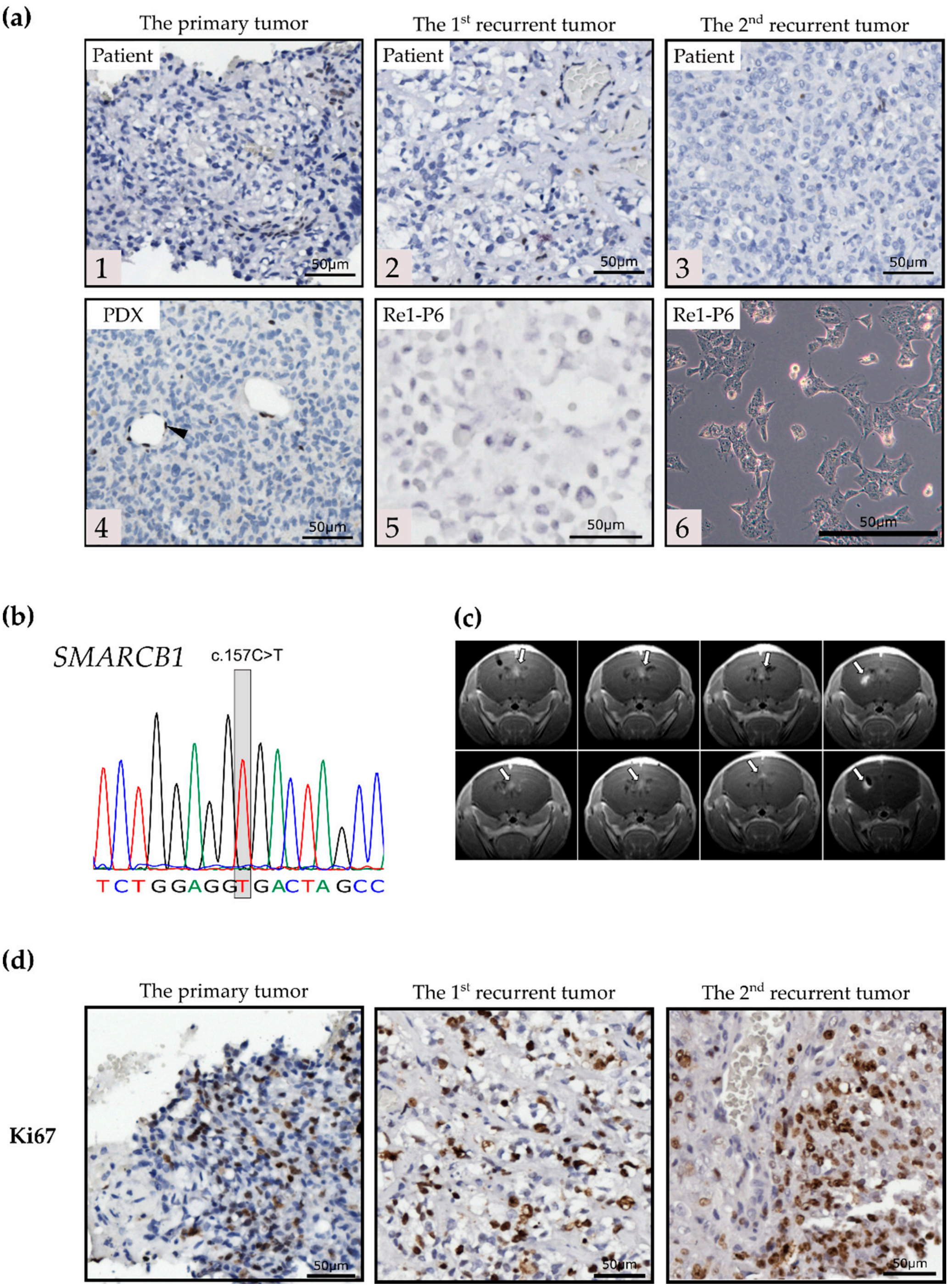

Figure S1. ATRT animal models and PDX-derived tumor cell line Re1-P6. **(a)** Immunohistochemistry staining for INI1 in patient samples (a1-3), the 6th passage of PDX sample (a4) and Re1-P6 cells (a5); Loss of INI1. Morphology of Re1-P6 cells was visualized as adherent cells by contrast-phase microscope with magnification x 10 (a6). Vascular endothelial cells were used as a positive control (black arrow) in A4. Scale bar, 50 µm. (**b**) Sanger sequencing confirmed the c.157C > T mutation in *SMARCB1* in Re1-P6 cells. (**c**) Post-contrast brain MRIs performed at 21 day-post transplantation showed the presence of tumors (n = 8). (**d**) IHC for Ki67 in the patient ATRT samples. Ki67 is upregulated in recurrent patient samples. Scale bar, 50 µm.

(a)

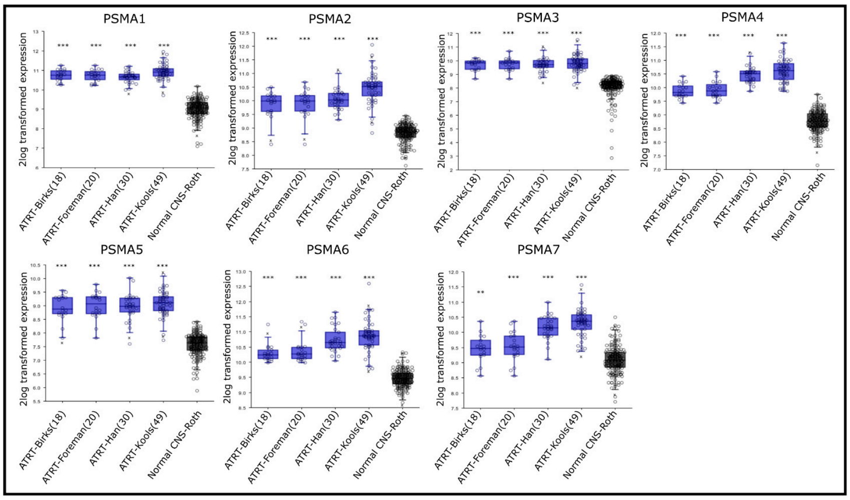
PSMA1
PSMA2
PSMA3
PSMA4
PSMA5
PSMA6
PSMA7
2log transformed expression
ATRT-Birks(18)
ATRT-Foreman(20)
ATRT-Han(30)
ATRT-Kools(49)
Normal CNS-Roth

(b)

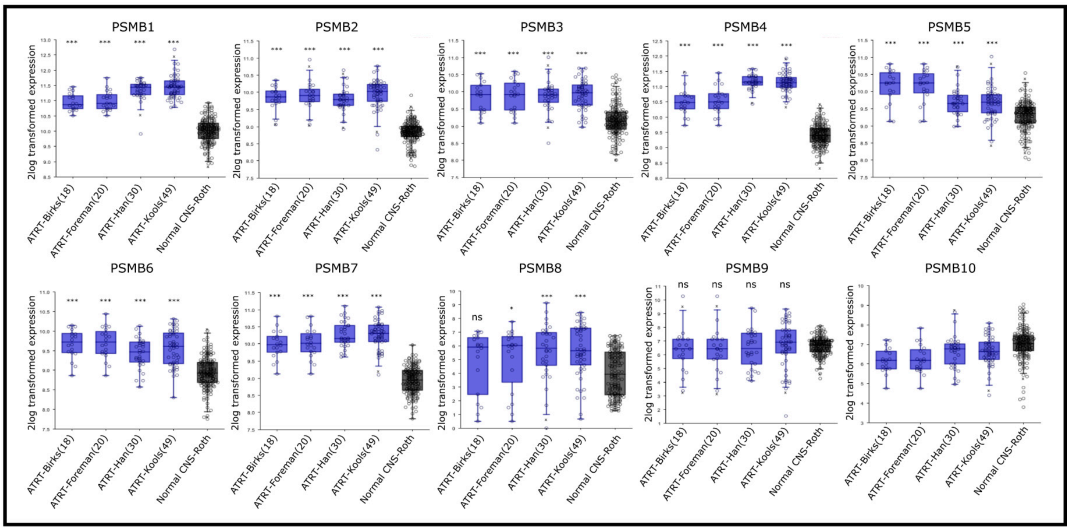
PSMB1
PSMB2
PSMB3
PSMB4
PSMB5
PSMB6
PSMB7
PSMB8
PSMB9
PSMB10
2log transformed expression
ATRT-Birks(18)
ATRT-Foreman(20)
ATRT-Han(30)
ATRT-Kools(49)
Normal CNS-Roth

(c)

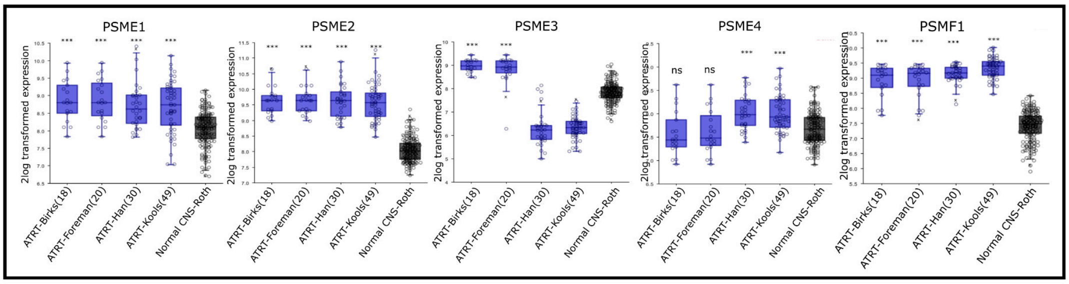
PSME1
PSME2
PSME3
PSME4
PSMF1
2log transformed expression
ATRT-Birks(18)
ATRT-Foreman(20)
ATRT-Han(30)
ATRT-Kools(49)
Normal CNS-Roth

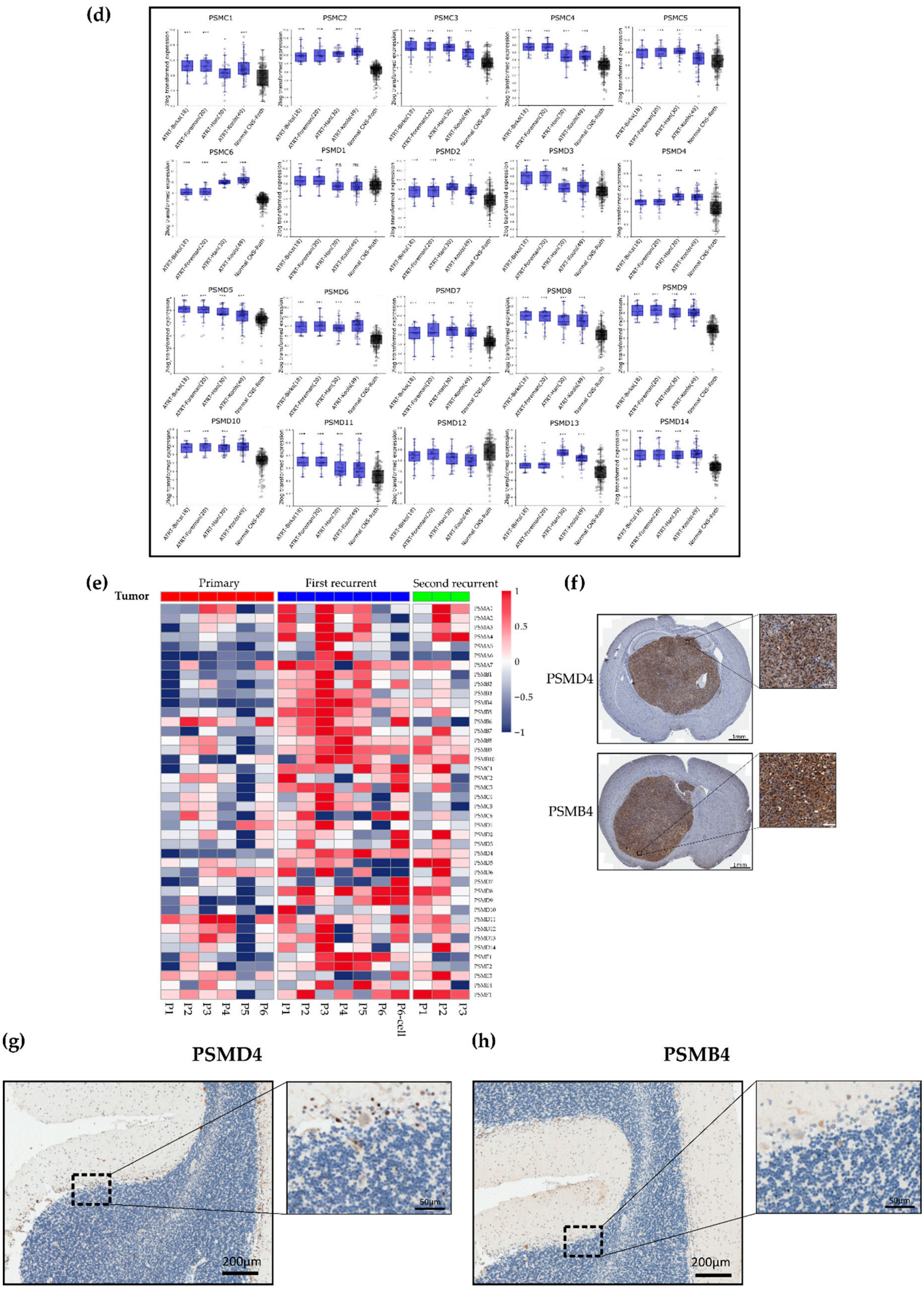

Figure S2. Overexpression of proteasome encoding genes in ATRTs. (**a**–**d**) The expression level of encoding genes of 20S proteasome (a,b), 11S proteasome (c), and 19S (d) in ATRT public data set and normal brain tissues, using the R2 Platform (http://r2.amc.nl). Most of the encoding genes of 20S proteasome (excluded *PSMA8*, *PSMB9*, and *PSMB10*), 11S, and 19S (excluded *PSMD12*) were overexpressed in ATRTs as compared with normal brain tissues. * $p < 0.05$, ** $p < 0.01$, *** $p < 0.001$, ns:

nonsignificant; P values are downloaded from R2 Platform (One-way analysis of variance test). (**e**) The heatmap of mRNA expression levels of the proteasome encoding genes in PDX samples. Proteasome encoding genes expressions are higher in recurrent PDX samples and Re1-P6 cells than in the primary PDX samples. (**f**) IHC staining for PSMD4 and PSMB4 in orthotopic xenograft mice from Re1-P6. High expression of PSMD4 and PSMB4 in brain tumors. (**g,h**) IHC staining for PSMD4 and PSMB4 in normal brain tissues. Low expression of PSMD4 and PSMB4 was found in normal brain tissue. Scale bar, 200 µm, 50 µm.

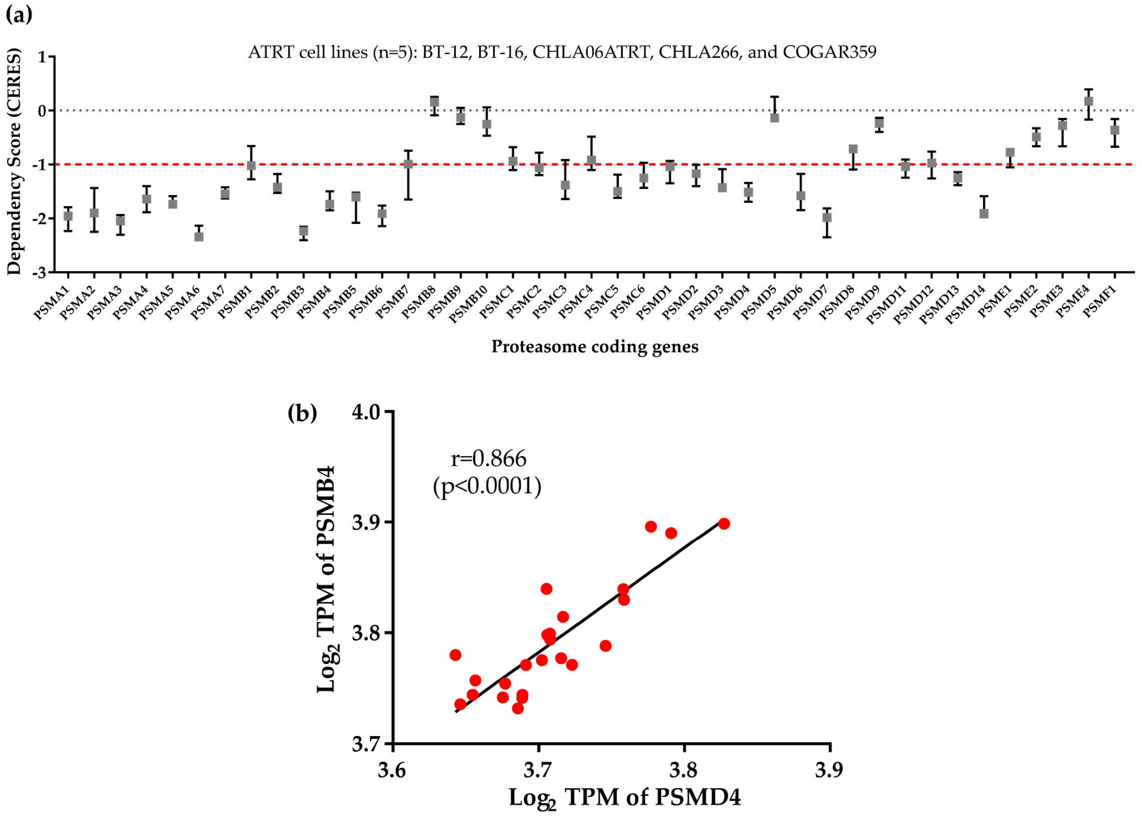

Figure S3. Proteasome encoding genes are essential for ATRT cells survival. **(a)** Dependency score of proteasome encoding genes in 5 ATRT cell lines (BT-12, BT-16, CHLA06ATRT, CHLA266, and COGAR359), Median with 95% CI. The data was download from project Achilles (CRISPR/Cas9 libraries in genome-scale pooled loss-of-function (LOF) screening) DepMap Public 19Q2 dataset [22]. 37 in 42 proteasome encoding genes have the dependency score less than 0. A gene is considered to be essential for a given cell line if the dependency score is less than 0. The lower dependency score means that the gene has a higher contribution to cell survival. The red dash line at score of -1 represents the median of all common essential genes. (**b**) High correlation between *PSMD4* & *PSMB4* in human ATRTs ($n = 24$), $r = 0.866$, $p < 0.0001$. Transcripts Per Kilobase Million (TPM) of *PSMD4* and *PSMB4* from RNA-seq were normalized using logarithm base 2.

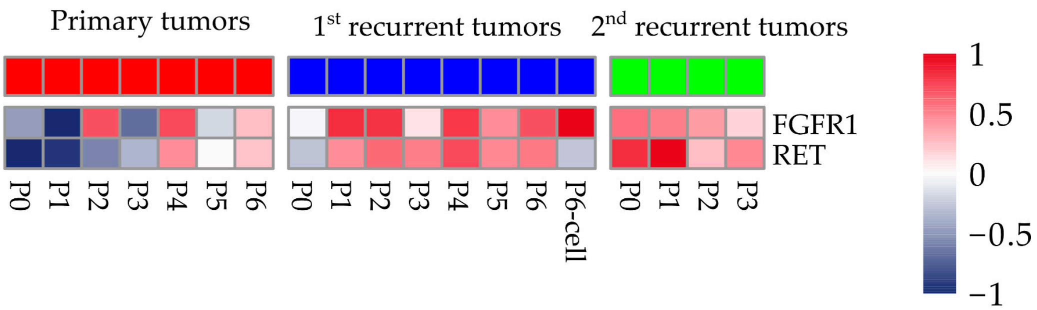

Figure S4. RNA Heatmap of *FGFR1* and *RET* expression in the tumor samples from the patient, PDX and Re1-P6 cells. Upregulation of *FGFR1* and *RET* in recurrent tumors.

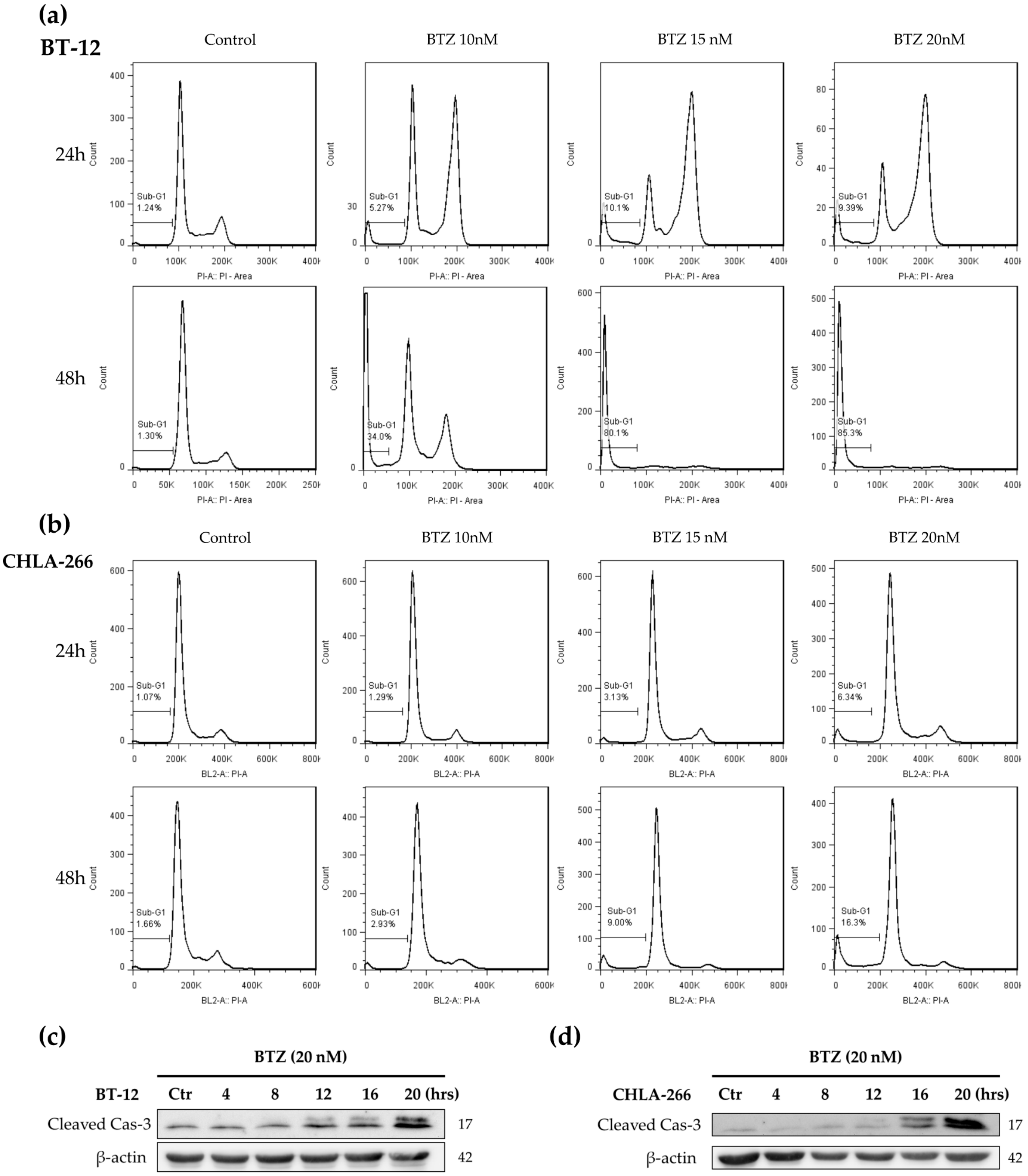

Figure S5. BTZ induced apoptosis in Myc-ATRT cells. (**a**,**b**) Flow cytometry analysis of BT-12(a) and CHLA-266(b) after being treated with BTZ. BT-12 and CHLA-266 were incubated with BTZ with three concentrations of 10,15,20 nM for 24 and 72 h, then fixed and stained with PI for cell cycle accessing by flow cytometry. BTZ increased the sub-G1 population of the treated cells in a dose-dependent and a time-dependent manner. (**c**,**d**) Immunoblotting for cleaved caspase 3 in BT-12 (c) and CHLA-266 (d) after incubation with BTZ. BT-12 and CHLA-266 were incubated with 20 nM BTZ for indicated times. The whole-cell lysate was resolved by SDS-PAGE and blotted with anti-cleaved-caspase-3 antibodies. BTZ induced the accumulation of Cleaved Caspase 3 in BTZ-treated cells.

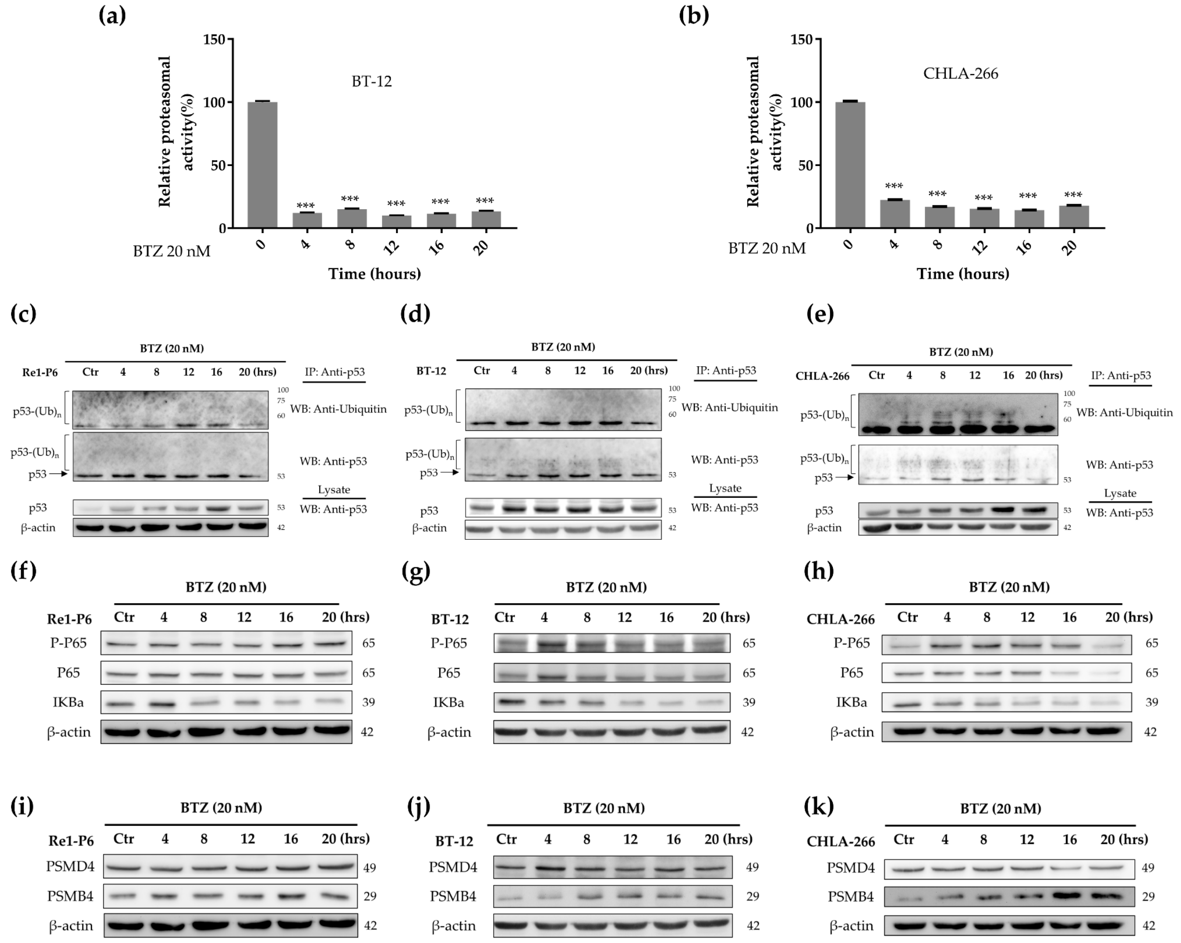

Figure S6. BTZ inhibited proteasome activity, resulting in the accumulation of P53 but not IκBa. (**a**,**b**) BTZ inhibited proteasome activity in BT-12 (a) and CHLA-266 (b). The whole-cell lysate was extracted from BT-12 and CHLA-266 after being incubated with BTZ (20 nM) for indicated times. 40 µg protein sample was incubated with substrate LLVT-AMC at 37 °C for 2 h. The released AMC fluorescence was quantified using a 380/460 nm filter. *** $p < 0.001$, t-test. The data represent the mean ± standard deviation ($n = 3$). (**c**–**e**) BTZ increased the accumulation of p53 and ubiquitinated p53 in Re1-P6 (c), BT-12 (d), and CHLA-266 (e). After incubation of BTZ (20 nM) for indicated times (from 4 to 20 h) The whole-cell lysate was prepared as previously described. Ubiquitinated proteins were immunoprecipitated by anti-p53 antibody, resolved by SDS-PAGE, and blotted for anti-ubiquitin antibody (Upper panels). The extracted proteins were immunoblotted for anti-P53 (Lower panels). (**f**–**h**) Immunoblotting for anti-phosphorylated p65, anti-p65, and anti-IκBa antibodies. Down-expressing IκBa was found in BTZ-treated cells, including Re1-P6 (f), BT-12 (g), and CHLA-266 (h). (**i**–**k**) Immunoblotting for anti-PSMD4 and anti-PSMB4 antibodies. BTZ did not downregulate the PSMD4 and PSMB4 expression in BTZ-treated cells, including Re1-P6 (i), BT-12 (j), and CHLA-266 (k).

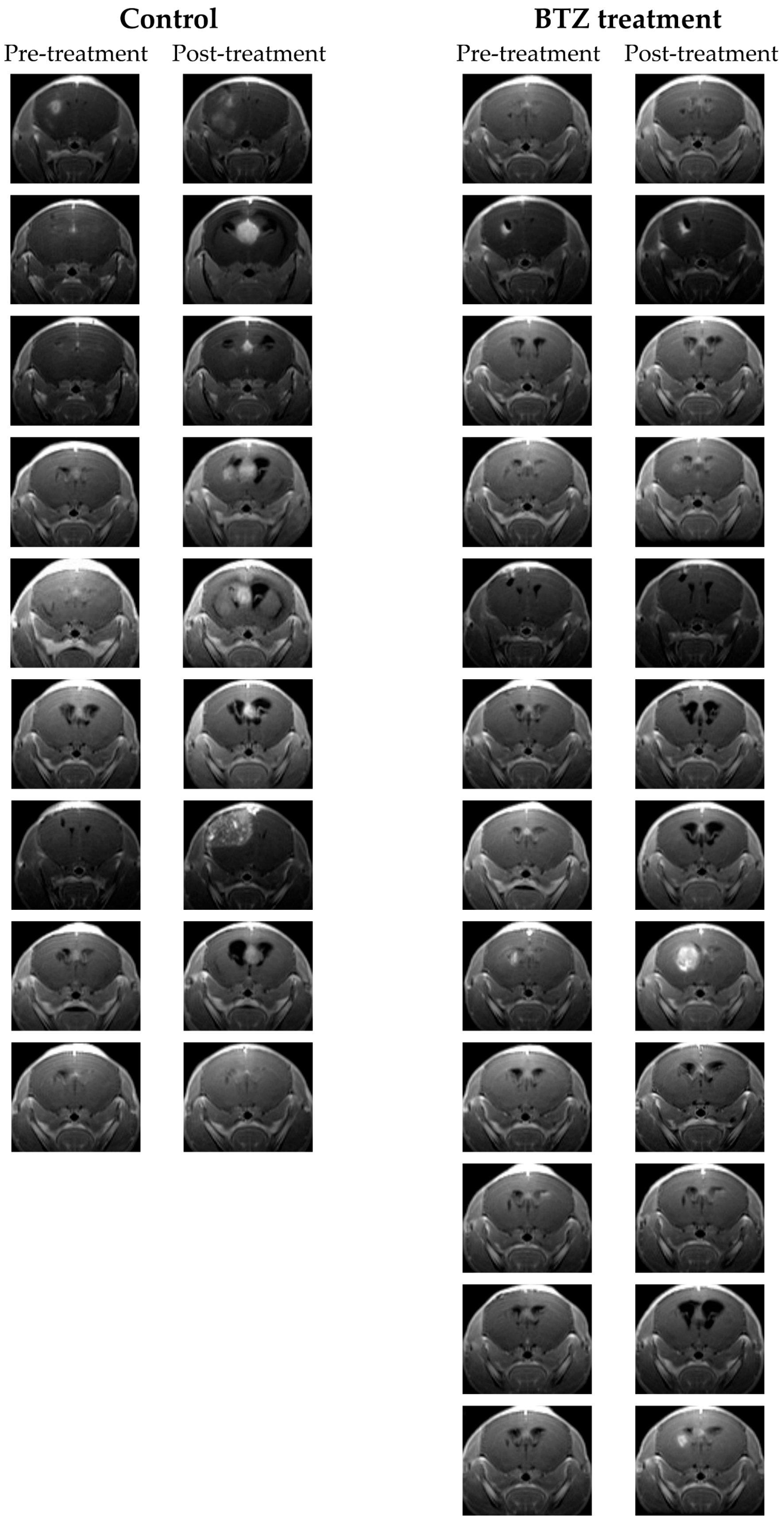

Figure S7. Pre-treatment and Post-treatment brain MRI in BTZ-treated group and control-group, related to Figure 5C,D. BTZ inhibited tumor growth in treated-group.

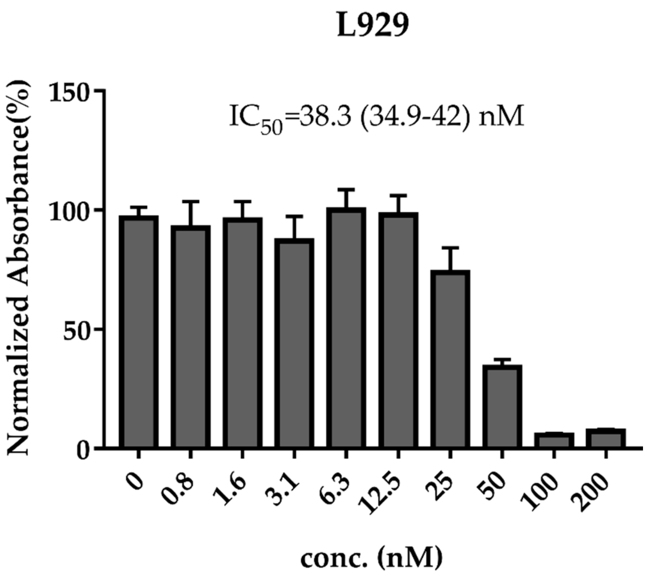

Figure S8. Cytotoxiciy effect of Bortezomib (BTZ) on L929 cell lines. MTT assay was assessed after 72 h of exposure to a serial dilution of BTZ (0.8–200 nM). The IC_{50} of of BTZ againt L929 was 38.3 (34.9–42) nM.

Table S1. Patients list of ATRT, related to Figure 2E, 6B, 6C, and S3B.

ID#	Gender	Age (years)	Subgroup	IHC of INI1	*SMARCB1* mutation	*SMARCA4* mutation	*TP53* mutation
T2605	M	0.7	SHH	(-)	Deletion	(-)	(-)
T480	M	0.04	SHH	(-)	(-)§	(-)	(-)
T3007	M	1.3	SHH	(-)	Stop-gain§	(-)	(-)
TM34	M	1.9	SHH	(-)	Frameshift deletion	(-)	(-)
T4280	M	0.9	SHH	(-)	Stop-gain	(-)	(-)
T2926	F	1.9	SHH	(-)	Stop-gain	(-)	(-)
TM71*	M	0.7	SHH	(-)	Stop-gain	(-)	(-)
RT1423	M	1.1	SHH	(-)	Stop-gain	(-)	(-)
T483	F	2.3	SHH	(-)	(-) §	(-)	(-)
T1545	M	1.6	TYR	(-)	(-) §	(-)	(-)
RT967	F	1.4	TYR	(-)	Deletion	(-)	(-)
T2852	F	1.1	TYR	(-)	Stop-gain	(-)	(-)
T4063	F	1.7	TYR	(-)	Deletion	(-)	(-)
T475	F	4.4	TYR	(-)	(-) §	(-)	(-)
T2367	M	1.5	TYR	(-)	Frameshift deletion§	(-)	(-)
T2318	F	0.6	TYR	(-)	Frameshift deletion	(-)	(-)
T2305	M	5	TYR	(-)	Frameshift deletion	(-)	(-)
T902	F	5.2	TYR	(-)	Frameshift deletion	(-)	(-)
T2750	M	1.6	TYR	(-)	Stop-gain	(-)	(-)
T4293	F	1.2	MYC	(-)	Deletion	(-)	(-)
RT1671	M	1.8	MYC	(-)	(-) §	(-)	(-)
T4128	F	0.6	MYC	(-)	Frameshift deletion	(-)	(-)
TM113 *	M	1.1	MYC	(-)	Stop-gain §	(-)	(-)
TM131 *	M	1.6	MYC	(-)	Stop-gain §	(-)	(-)

* TM71, TM113, and TM131 are the ID numbers of the primary, the first recurrent, and the second recurrent samples of one patient, respectively. § The results were identified based on RNA-Seq data. IHC: Immunohistochemistry.

Table S2. Information about normal brain tissues.

GEO accession	Tissue	Subject status	Affiliates
GSM2501173	Normal brain	Pediatric brain tumor patient	Jabado Lab, Department of Pediatrics, McGill University
GSM2501174	Normal brain	Pediatric brain tumor patient	Jabado Lab, Department of Pediatrics, McGill University
GSM2501175	Normal brain	Pediatric brain tumor patient	Jabado Lab, Department of Pediatrics, McGill University
GSM2193194	Normal brain	Fetus	Center for Pharmacogenomics, School of Life Sciences, Fudan University

Table S3. IC_{50}-IC_{90} of Bortezomib, Alisertib, SAHA, and Lenvatinib in Re1-P6, BT-12, and CHLA-266 cells.

Drugs	Cell lines	IC_{50} (μM)	IC_{90} (μM)
BTZ	CHLA-266	0.007	0.016
	BT-12	0.006	0.011
	Re1-P6	0.009	0.014
Alisertib	CHLA-266	7.7	558.0
	BT-12	0.07	276.9
	Re1-P6	0.63	41.3
SAHA	CHLA-266	10.5	822.9
	BT-12	3.9	24.2
	Re1-P6	6.3	66.8
Lenvatinib	CHLA-266	19.8	1242.1
	BT-12	50.2	179.2
	Re1-P6	1.5	67.7

IC: Inhibitory concentration

© 2020 by the authors. Licensee MDPI, Basel, Switzerland. This article is an open access article distributed under the terms and conditions of the Creative Commons Attribution (CC BY) license (http://creativecommons.org/licenses/by/4.0/).
